# Continuous shear stress alters metabolism, mass-transport, and growth in electroactive biofilms independent of surface substrate transport

**DOI:** 10.1038/s41598-019-39267-2

**Published:** 2019-02-22

**Authors:** A-Andrew D. Jones, Cullen R. Buie

**Affiliations:** 1 Department of Mechanical Engineering, Massachusetts Institute of Technology, Cambridge, MA 02139 USA; 2 Present Address: Department of Chemical Engineering and Department of Mechanical & Industrial Engineering, Northeastern University, Boston, MA 02115 USA

## Abstract

Electroactive bacteria such as *Geobacter sulfurreducens* and *Shewanella onedensis* produce electrical current during their respiration; this has been exploited in bioelectrochemical systems. These bacteria form thicker biofilms and stay more active than soluble-respiring bacteria biofilms because their electron acceptor is always accessible. In bioelectrochemical systems such as microbial fuel cells, corrosion-resistant metals uptake current from the bacteria, producing power. While beneficial for engineering applications, collecting current using corrosion resistant metals induces pH stress in the biofilm, unlike the naturally occurring process where a reduced metal combines with protons released during respiration. To reduce pH stress, some bioelectrochemical systems use forced convection to enhance mass transport of both nutrients and byproducts; however, biofilms’ small pore size limits convective transport, thus, reducing pH stress in these systems remains a challenge. Understanding how convection is necessary but not sufficient for maintaining biofilm health requires decoupling mass transport from momentum transport (i.e. fluidic shear stress). In this study we use a rotating disc electrode to emulate a practical bioelectrochemical system, while decoupling mass transport from shear stress. This is the first study to isolate the metabolic and structural changes in electroactive biofilms due to shear stress. We find that increased shear stress reduces biofilm development time while increasing its metabolic rate. Furthermore, we find biofilm health is negatively affected by higher metabolic rates over long-term growth due to the biofilm’s memory of the fluid flow conditions during the initial biofilm development phases. These results not only provide guidelines for improving performance of bioelectrochemical systems, but also reveal features of biofilm behavior. Results of this study suggest that optimized reactors may initiate operation at high shear to decrease development time before decreasing shear for steady-state operation. Furthermore, this biofilm memory discovered will help explain the presence of channels within biofilms observed in other studies.

## Introduction

Bacteria exist in biofilms more than in planktonic states to (1) protect bacteria from predation and chemical attack and (2) to allow bacteria to manipulate their environment^1^. Electrochemically active biofilms^2–4^ not only have potential to bridge the energy-water nexus, producing power from wastewater treatment^5,6^, but also to be used as a tool to study biofilm development because they produce a measurable current as part of their metabolism^2^. Though bioelectrochemical systems employ flow to improve nutrient delivery and metabolic waste removal^6–9^, which is known to influence biofilm behavior, the independent influence of shear on electrochemically active biofilms remains largely unexplored^6–11^. Short-term exposures of electroactive biofilms to shear showed expected improvements in salt transport^12^ and unexpected resilience to high shear (635 s^−1^)^13^ despite forming thicker biofilms than aerobic bacteria^5^. Long-term exposures to shear showed that high shear (200 s^−1^) selects for high-current producing bacteria compared to low shear (80 s^−1^)^14^. That fluid shear affects bacteria biofilms fixed to a surface and that biofilms have channels^15^ is not well understood because pore sizes limit convection^16–19^ and thus the influence of shear. Electroactive bacteria biofilms remain more metabolically active^20,21^ than soluble respiring bacteria and might not be limited by respiration in high shear unlike aerobic biofilms^22^. Using the analytic equations for fluid flow and mass flux that exist for a rotating disk electrode, the absolute concentration of nutrients is reduced in a way that decouples the influence of mass flux and shear stress. We use a pure culture of a high-current producing bacteria to reduce the effect shear has on selection of species with high adhesion strength, exopolymeric substance production, or current production. However, there will still be phenotypic selection within the strain induced by time and possibly shear^23^. In doing so, we find high metabolic rate increases with increased shear that may improve the performance of bioelectrochemical systems during startup. We find two dimensionless groups that account for initial, exponential and maximum growth though not decay. We demonstrate that while these biofilms are affected by their environment, their porosity does not change at steady-state so they are still susceptible to decay from pH stress^5^. This paper has been published as a BioRxiv pre-print^24^, is extended from A. Jones’ dissertation^25^, and has been presented in part at previous meetings^26^.

We used a rotating disk electrode that has analytic equations for flow and mass flux that can be varied independently, and through scaling laws, have been used to simulate pipe flow, conduit flow and spray jets^27^. The property of separation, specifically, makes rotating disk electrodes useful for studying electrochemical reactions; however, when it has been used to study electroactive biofilms in the past, this particular property was not used^28^. We benchmarked our initial flux and shear to that of a practical bioelectrochemical system^6^, and varied the shear by two orders of magnitude. We used a pure culture of *Geobacter sulfurreducens*, as it has been shown to produce the highest current^5^ in a bioelectrochemical system and is the most abundant species in mixed-culture bioelectrochemical systems when selected through time, shear, or voltage methods.

## Results and Discussion

**Figure 1:**
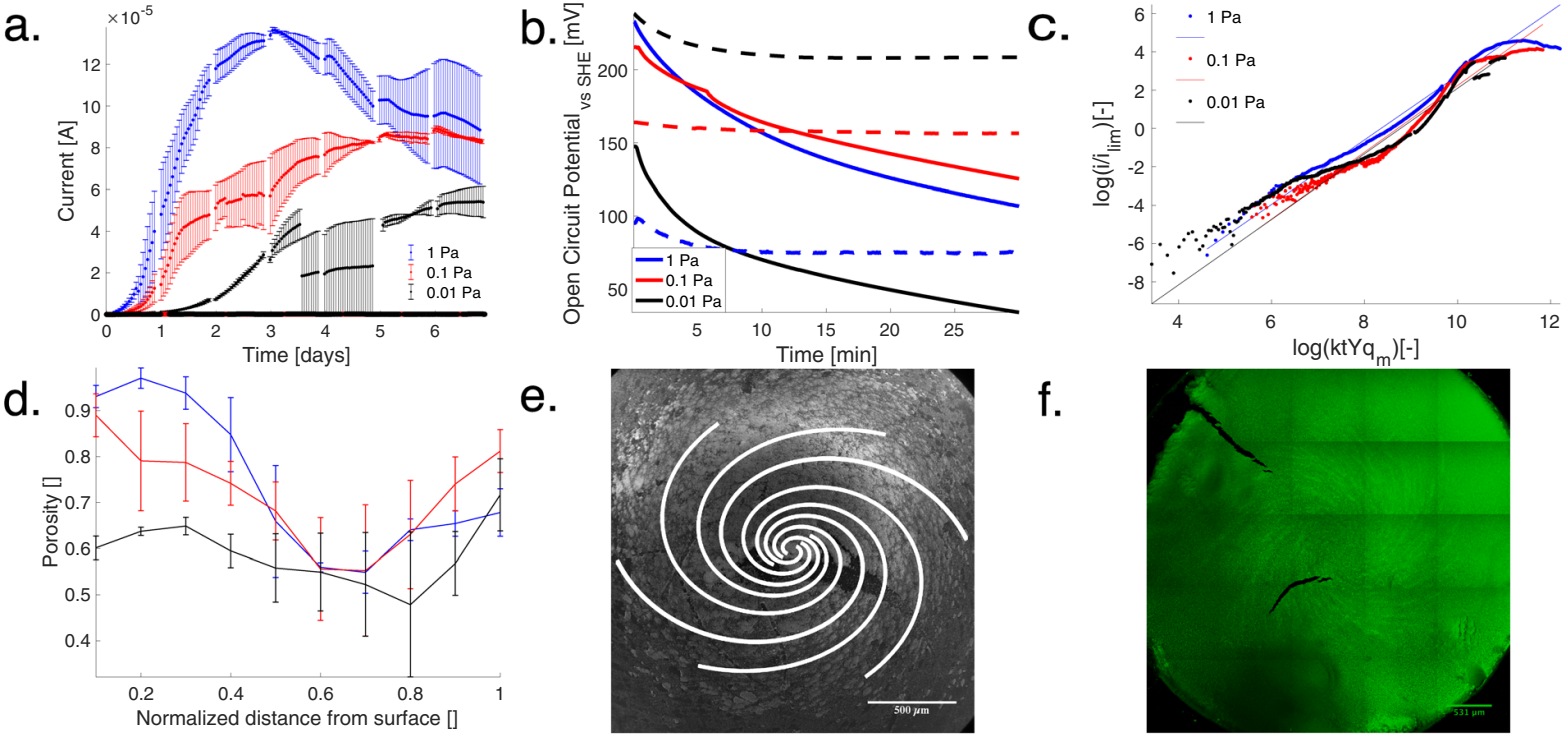
(**a**) The current produced by *G*. *sulfurreducens* at three shear stresses of 1 Pa, 0.1 Pa (*s*.*e*. *n* = 3) and 0.01 Pa (*s*.*e*. *n* = 2). The abiotic measurements never exceed 10^−7^ A for each shear stress condition. The flux was fixed to an up-flow microbial fuel cell (He *et al*.^6^) which also corresponded to the 1 Pa shear stress case. We find that at the lower shear stress, the maximum current persists longer. The maximum current was 136 ± 1.24 µA, 89.3 ± 1.24 µA and 54.03 ± 7.08 µA at 1 Pa, 0.1 Pa and 0.01 Pa in blue, red and black respectively. (**b**) Time-averaged open circuit potential for the first 30 minutes after inoculation shows decreasing potential with bacteria present in contrast to the stable ‘--’ lines of the abiotic control. (**c**) Dimensional analysis reveals two parameters of interest a scaled growth time Eq. 2 and a ratio of current to maximum electrons delivered, Eq. 1. These parameters are fit to a dimensionless model, showing that the lowest shear case has not yet reached the maximum metabolic current output as would be predicted by scaling and would be anticipated from (**a**). This model does not account for the decrease in current. (**d)** Porosity of the biofilm as a function of distance from the surface normalized to the height obtained from confocal microscopy of fixed biofilms immersed in ethanol (**f**). This data shows that the biofilms in the 1 Pa and 0.1 Pa cases, which we predict as fully-developed, have similar structure, whereas the 0.01 lowest shear case does not. (**e**) An image of the biofilm from an underperforming 1 Pa shear stress condition (16 µA after 7 days). The biofilm shows a growth pattern similar to the streamlines predicted by the von Kármán solution to flow at a rotating disk. (**f**) A confocal image slice 6 µm from the surface of an electroactive biofilm taken after 7 days at 0.1 Pa shear stress, showing the predicted streamlines of the fluid flow which should not be present ~34 µm into the biofilm. We assume this is “memory” of flow-influenced adhesion.

We find that maximum current and rates of current increase follow increasing shear stress for continuous growth of *G*. *sulfurreducens* PCA with fixed mass flux to the surface of a rotating disk, Fig. 1a. The rise in current follows a logistic growth like pattern from slow initial rise, exponential increase to maximum followed by a decay. Increasing shear stress from 0.01 Pa to 1 Pa reduced the initial doubling time of the current from 0.26 days to 0.14 days, as shown in Table 1 and Supplementary Figure S1. The near immediate current response is found in the literature^29^, but here we also found a dependence on shear stress. We propose that the rapid development of measurable current is also independent of the flux of bacteria, which was not held constant. If the variable flux of bacteria caused the rapid current development, it would be reflected in the initial average open circuit potential (OCP) which is used to describe active bacteria colonization of an electrode^30^. However, the OCP shows a decay rate (Table 1, Fig. 1b) that is inconsistent with the trend in current rise. We assume that all the bacteria had the same initial electrochemical activity, as they were all taken from mid-log planktonic growth phase^21^ and seeded at 600 RPM for 10 minutes prior to the start of each experiment. However, the variability shown in Fig. 1b demonstrates that further study of the influence of bacteria on electrode potential is needed^30^.

**Table 1:** Growth and current parameters show linear dependence on shear stress.

Shear [Pa]	OCP drop [mV/min]	*t*_*1/2*_ [days]	*t*_*max*_ [days]	*I*_*max*_ [µA]
1	4.2	0.14	3.8	136 ± 1.24
0.1	3.0	0.12	4.3	89.3 ± 1.24
0.01	3.8	0.26	9.8	54.3 ± 7.08

There are linearly increasing trends in doubling time and time to maximum current with increased shear, and linearly decreasing trends in maximum current. The time, *t*_*max*_, to maximal growth is estimated using a dimensionless model based on logistic-growth. The doubling time of current, *t*_*1/2*_ estimated assuming an exponential rise over the first 24 hrs.

We used dimensional analysis to estimate that the time to maximum current is decreased for increased shear rates from 9.8 days to 3.8 days, as shown in Fig. 1c and Table 1. The electrochemical signal of the biofilm displayed similar characteristics to optical measurements of planktonic cell growth^31^ and optical measurements of biofilm growth^32^. Assuming that current is a proxy for metabolic rate^2^, the initial lag phase, where little current is found, was seen in the 0.01 Pa case (Fig. 1a). A rapid growth phase can be seen in all three shear rates tested, a stable growth phase can be seen in the 0.1 Pa case, and a decay phase can be seen in the 1.0 Pa case. Thus, we assumed that the dimensionless current should scale with dimensionless time according to a logistic growth model.

**Figure 2:**
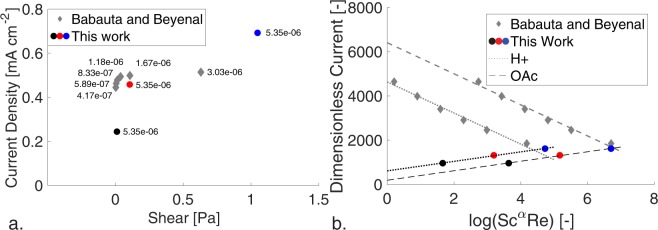
(**a**) A comparison of shear stress and current found in our work with that found by Babauta and Beyenal using the same species, electron donor, and reactor dimensions. The flux in our work, 5.35e-6 g COD cm^−2^ s^−1^, is strictly greater than their fluxes tested however the current results vary. (**b**) Dimensionless current against the product of the Schmidt number and Reynolds number shows linear dependence of biofilm metabolism on viscous shear and mass transport. The Schmidt number goes as α = 1 for both acetic acid and proton transport in this work, with *R*^2^ = 0.99. The Schmidt number goes as α = −1 for acetic acid and α = 1.1 for proton transport for Babauta and Beyenal’s work, with *R*^2^ = 0.98.

Our results indicate a shear dependence on maximum current not described in the literature^28^ when both mass and shear were coupled (Fig. 2a.) for the same species. It is accepted that varying concentration (mass flux) changes metabolic (current) output for all bacteria, and varying electrode potential^33^ and electric fields^34^ can improve power output and select for electroactive biofilms. From this hypothesis, previous studies have grown the biofilm under one shear stress condition and varied shear to see the impact on current^28^. A common technique for determining the influence of shear, substrate convection, and substrate diffusion is the use of the product of the Schmidt and Reynolds number, where the Schmidt number is a ratio of viscous diffusion to mass diffusion, Sc = *v*/*D*, to scale mass transport and the Reynolds number is a ratio of the inertial to viscous forces, Re = *uD*/*v*, to scale shearing forces. Combining this product and the scaling of current from Fig. 1c, we find different metabolic efficiencies and transport regimes for biofilms subjected to shear from different starting conditions determined by a parameter *α*, as $i/{i}_{\mathrm{lim}}\sim {{\rm{Sc}}}^{\alpha }{\rm{Re}}$. The maximum current of biofilms initially grown under shearing or static conditions, 136 µA and 101 µA respectively, becomes the same when scaled and occurs at the same scaled transport condition, Fig. 2b. Furthermore, these points coincide whether using the diffusivity of acetate (substrate) or protons (toxic waste byproduct), Fig. 2b. However, biofilms grown in static conditions show a decrease in dimensionless current with increasing transport. Our dimensionless current is equivalent to the Coulombic efficiency, so we hypothesize that the biofilm is adapted to maximum utilization at its initial growth condition (shear stress of 0 Pa) and any increase does not improve substrate utilization. This adaptation is likely the higher porosity found for biofilms not grown under shear^35^. For both, protons or acetate, biofilms in this work are strictly dependent on mass transport, α = 1. However, biofilms initially grown in static conditions are dependent on both diffusivity and viscosity for acetate transport α = −1 and weakly dependent on viscosity α = 1.1 for proton transport. Since no hysteresis in current output was found for the biofilms grown in static conditions and then sheared indicating no loss of biomass^28^, this dependence on viscosity should be further investigated.

High metabolic rates described appear to come at the cost of sustained growth and stable metabolic activity. The highest shear stress, 1.0 Pa, produced the highest current density (see Table 1) yet immediately declined (Fig. 1a). This decline in current was not present for the lower shear stress cases. While it has been shown that higher shear induces higher metabolic rates in non-electroactive biofilms^36^, previous experiments were run for half the duration of the present study. As a result, they did not show the trade-offs found here-in for the highest shear^36^. We caution that our dimensional model, Fig. 1c, does not describe the decay shown in the high shear stress case, though it does help determine when it may be necessary to run experiments for longer durations^1^.

Measured electron diffusion current followed similar trends as the electrical current with shear (Supplementary Fig. S6.). Since electron diffusion current is dependent on electron transport protein concentration, bacteria concentration, and reaction rate^37^, and high current production^5^ and/or high metabolic activity^32^ leads to decreased pH in the biofilm with a concomitant loss of electron transport protein activity^5^, we hypothesize that the decline is due to either the death of bacteria or the loss of electron transport protein activity. Nyquist plots, Supplementary Fig. S3, of the electrochemical impedance spectroscopy, a measure of the complex resistance to charge transfer, similarly showed an increase in resistance upon a decrease in current (both electron diffusion and absolute), which could either imply cell death or loss of electron transport protein activity. To interpret this further impedance spectra measured at 340 mV were fit using a simple equivalent circuit model^38^. The charge transfer resistance shows a nonlinear decay for the lower shear cases and a nonlinear increase for the highest shear. The pseudocapacitance shows an oscillation about a mean time. These two features do not explain the overall trends in current or the full impedance. However, using an apparent diffusivity calculation for the substrate acetate following Tribollet *et al*.^39^, shows steady exponential decay for the highest shear, and a crossover for the lowest contrast with the electron diffusivity (Supplementary Fig. S6). Taken together, there are likely internal regulations of substrate and electron transport similar to those found in static conditions on pH and turnover conditions that must be further investigated with continuous structural measurements to produce the external results shown here.

While biofilm health is negatively affected by higher shear stress over long-term growth, the biofilm retains memory of initial fluid flow conditions and does not change structure to counteract such stress, Table 2. The independence of thickness and shear for the two higher shear stress cases agrees with previous studies on membrane aerated biofilms^10^. Since the lowest shear stress case has not reached maximal current, we conclude that biofilm thickness, surface roughness, and porosity are independent of shear for the conditions tested once the biofilm is fully developed (Fig. 1d).

**Table 2:** Similarities in thickness (H) and surface roughness (*R*_*a*_) appear to confirm that the 1 and 0.1 Pa cases biofilms are fully developed while the 0.01 Pa case is still developing. *n* = 2.

Shear [Pa]	H[µm]	*R*_*a*_
1	41	2.50
0.1	41	2.07
0.01	16	1.36

The fluid streamline pattern (Fig. 1f) is imprinted on the biofilm interior at a height of 6 µm, but not at the surface of the biofilm after seven days of growth. The stability of this interior structure is quantitatively seen in stable open circuit potential^30^ and redox potential^37^, indicating a stable surface attachment of electrically respiring bacteria/proteins, Supplementary Figures S4–S5. The mechanisms of this imprinting were not studied, though it may be due to the transport of quorum sensing chemicals during biofilm formation. The memory of a complex substructure from initial growth conditions of a biofilm has been seen before^15^, yet this is the first time to the authors’ knowledge that it has been shown under controlled initial conditions.

This analysis does not account for additional coupling of growth factors. Imposing shear stress and electric field gradients^34^ on mixed species biofilm in long-durations has been shown to select for electroactive bacteria. When decoupled from the impact of mass transport, long-time exposure to shear may show genetic adaptation in structure and current generation^40^. Further tradeoffs between EPS production and current generation may be affected by shear^22^. We propose that the linear relationship of dimensionless current to dimensionless biofilm growth rate, Fig. 1c, would be improved with data showing transport through the biofilm. This could be in the form of pH monitoring, acetate utilization, or continuous structural monitoring as described. In comparing with the results Babauta and Beyenal^28^, electrode potential was not fixed as the present study used the recommendation for maximum current production from Soussan *et al*.^41^. The coincident dimensionless current warrants further study as potential has a strong effect on current production^34,41^.

## Conclusion

This study shows that electroactive biofilms increase metabolic activity in response to increasing shear stress independent of mass transport. This occurs both at early times and at long-times. Using a dimensionless model, we show initial growth conditions can shift the system from diffusion dependent to convection dependent mass transport by comparing with results from the literature. However, this increased metabolism comes at the cost of decreased viability, likely due to an inability to transport metabolic byproducts. This result is consistent with the existing literature suggesting that shear stress does not induce changes in biofilm structure in both single and multi-species biofilms^10,42^. Continuous optical measurements and metabolic substrate monitoring may further improve understanding of the coupled phenomena and refine the fit between dimensionless growth and substrate utilization. These results are promising for optimizing bioelectrochemical system startup times as dynamic control of shear stress should be considered an independent tool for biofilms to maintain reactor stability similar to variation of potential^33^, batch feeding^43^, and removal of “old” biomass^44^.

## Methods

### Practical Benchmark

We benchmarked this study to an upflow microbial fuel cell by He *et al*.^6^. An upflow microbial fuel cell is designed in a similar manner to an upflow anaerobic sludge blanket bioreactor where organic laden fluid is flown upwards over a biofilm-coated porous media, having a smaller footprint than continuous stirred tank reactors and subsequently higher shear^45^. Matching two fluid flow systems, using only the dimensionless Reynolds number that is a ratio of momentum fluxes, produces vastly different results between the two systems^46^. Instead, both the mass flux and the momentum flux must be matched. The dimensionless shear stress for the upflow microbial fuel cell is found using Supplementary Eq. 5, $\mathop{\tau }\limits^{ \sim }=0.056$. This value is matched to the shear of rotating disc setups which for our system results in a rotation rate of 739 rpm, a shear stress of 1.048 Pa and shear rate of 1046 s^−1^. Using the data from He *et al*.^6^, and Supplementary Eq. 6 the flux can be found $N=5.35\text{e-6}\,{\rm{g}}\,{\rm{C}}{\rm{O}}{\rm{D}}\,{{\rm{c}}{\rm{m}}}^{-2}{{\rm{s}}}^{-1}.$ We varied the dimensional shear stress over two orders of magnitude keeping the mass flux fixed resulting in the concentrations, rotation rates, and dimensionless shear stress in Table 3.

**Table 3:** The experimental parameters used for an acetate-fed *G*. *sulfurreducens* rotating disk system.

Rotation Rate [RPM]	Shear [Pa]	Concentration [mM]	Dimensionless Shear []
739	1.048	14.97	0.0562
159	0.1048	32.24	0.1211
34	0.01048	69.47	0.2610

The top row is based on the experimental parameters of He *et al*., scaled as described herein^6^.

The current will be normalized to the mass transfer limiting current for a rotating disk,

1 $$\begin{array}{c}\frac{i}{{i}_{lim}}=\frac{i}{nFAN}=i{({n}_{e}FAG^{\prime} (0){\nu }^{-\frac{1}{6}}{\omega }^{\frac{1}{2}}{c}_{0}{D}_{0}^{\frac{2}{3}})}^{-1}\end{array}$$

To correlate this with the flux through the biofilm, the biofilm growth time is considered as

2 $$\mathop{t}\limits^{ \sim }=tY{q}_{m},$$

where *Y* is the biomass yield of the substrate and *q*_*m*_ is the maximum substrate utilization rate^47^. A plot of $\mathop{i}\limits^{ \sim }$ versus $\mathop{\tau }\limits^{ \sim }$ at constant mass flux results in curve that is similar to that of logistic growth^48^ with parameters that may relate to physical mechanisms.

### Media

The media was 0.59 g potassium dihydrogenphosphate, 0.38 g potassium chloride, 2.19 g sodium hydrogen carbonate, 0.36 g sodium chloride, 0.20 g ammonium chloride, 0.04 g calcium chloride dihydrate, and 0.10 magnesium chloride hexahydrate (MilliporeSigma, Darmstadt, GER) in 1 L of deionized water based on the media used in the literature^28,49^. The resulting conductivity was 2.10 mS.cm^−1^ at 30 °C. To this media was added 14.97 mM, 32.24 mM, or 69.47 mM of sodium acetate. This resulted in conductivity between 5.5 to 6.7 mS.cm^−1^. Acetate concentration was held constant by removing old media and adding gas sparged new media using a peristaltic pump at 0.07 mL. min^−1^.

### Electrodes

The working electrode was a 0.5 cm diameter glassy carbon exchange disk (Pine Instruments, Durham, NC, USA). The electrode was soaked in 1 M hydrochloric acid and 1 M sodium hydroxide for 24 hours after wiping with ethanol and roughened using 5 µm grit silicon carbide polishing paper to clean from previous experiments. A single junction Ag/AgCl (3.8 M KCl) reference electrode (MilliporeSigma, Darmstadt, GER) was used because the conductivity of the media is too low to sustain a double-junction. While this may cause leakage of the 3.8 M sodium solution into the electrolyte, the abiotic controls do not show any significant deviation. A 0.64 cm diameter graphite electrode behind a glass frit was used as the counter electrode (Pine Instruments, Durham, NC, USA).

### Apparatus

The reactor was a water jacketed inverted 500 mL Erlenmeyer flask, with 4 ports for electrodes and gas (Pine Instruments, Durham, NC, USA). The reactor was maintained at a constant temperature of 30 °C using a water jacket (Julabo E5, Julabo USA, Allentown, PA, USA). The system was purged and blanketed at 50 sccm with ultra-high purity 80% nitrogen and 20% carbon dioxide gas (Airgas Inc, Radnor Twp, PA, USA). The current was measured with a Gamry Reference 600 or a Gamry Reference 3000 (Gamry Instruments, Philadelphia, PA, USA) with a resolution of 600 mA and a bandwidth of 10 MHz which is much lower than the sampling rate and current measured.

### Bacteria

*Geobacter sulfurreducens* PCA (ATCC, 51573), was grown anaerobically from a frozen stock in a media matching Coppi *et al*. with 4 mM L-cystine and 40 mM fumarate^49^ at 30 °C. This was transferred to a 100 mL flask and grown for two days. The cells were centrifuged twice at 6000 rpm for 10 min, rinsed in between with the media described earlier. Optical density was measured at 600 nm (UV-1800, Shimadzu, Nakagyo-ku, Kyoto, JPN) after suspending the cells in the full 100 mL. Cell density was then calculated using a disposable hemocytometer (Incyto, Co., Ltd, Chonan-si, Chungnam-do, KOR). Cell volume was calculated assuming the bacteria were cylindrical and measuring them using a 100 X oil immersion objective.

### Procedure

The media was brought to 30 °C and internal resistance of the media was measured. All conditions were run in biological triplicate. A roughly 24 hr cycle included 30 min open circuit potential measurement, followed by two cycles of cyclic voltammetry, chronoamperometry for 22 hours, and electrochemical impedance spectroscopy. Chronoamperometry was conducted at −0.156 V vs Ag/AgCl based on Soussan *et al*.^41^. Open circuit potential was measured at a sampling rate of 0.016 Hz for 30 min as this appeared sufficient to reach steady-state. Cyclic voltammetry had an equilibration time of 5 s, a step size of 2 mV and a scan rate of 1 mV.s^−1^ from −0.755–0.045 V. following the method of Marsili *et al*.^38^. The electron diffusion current was measured as the maximal current generated during a slow (2 mV.s^−1^) cyclic voltammetry scan. Electrochemical impedance of the biofilm is measured since it may be related to the structure of the biofilm and is a key characteristic of microbial fuel cell performance^16,50,51^. Electrochemical impedance spectroscopy was conducted using two sets of parameters 1000000 Hz to 0.10 Hz at *E*_*dc*_ = −0.340 V, *E*_*ac*_ = 5 mV and 1000000 Hz to 0.01 Hz at *E*_*dc*_ = −0.157 V, E_ac_ = 10 mV following both Babauta and Beyanal and Marsili *et al*.^28,38^. We fit the impedance spectra to the equivalent circuit in Marsili *et al*.^38^, where the solution resistance is in series with the charge transfer resistance and a constant phase element in parallel. However, there is no widely accepted model for biofilm impedance. We further fit the impedance data by calculating an apparent diffusivity for acetate following the method of Tribollet *et al*.^39^ which holds when the system is mass transport limited.

### Optical Analysis

The biofilm density and thickness are necessary to compare the results to previous research on bioreactors^44^. After 7 days, the bacteria on disk were dyed using 4′,6-diamidino-2-phenylindole a DNA stain (NucBlue, Molecular Probes, Inc, Eugene, OR, USA) and Alexa Fluor® 594 -Concanavalin A, Conjugate (Molecular Probes, Inc, Eugene, OR, USA) that binds to glycocalyx^18^. After 10 minutes, the cells were fixed for 24 hrs using 4% SEM grade glutaraldehyde (MilliporeSigma, Darmstadt, GER) solution in their native buffer. The biofilm was then rinsed in an ethanol series 30, 50, 70, 90, 100, 100. These were imaged using confocal microscopy on four 50 µm^2^ regions at the perimeter of the disk where the shear was calculated. The sample was then critical point dried and sputter coated. The images were processed in MATLAB where a threshold was set and the images were converted to black and white. Porosity was calculated layer by layer.

## Supplementary information

Supplementary Information

## Data Availability

All data available upon request.
