# Supplementary material for: Continuous shear stress alters metabolism, mass-transport, and growth in electroactive biofilms independent of surface substrate transport
Source: Sci Rep. 2019 Feb 22;9:2602. doi: 10.1038/s41598-019-39267-2 (PMC6385357; doi:10.1038/s41598-019-39267-2)
Supplement: Supplementary file 1 — Supplementary Information [file 41598_2019_39267_MOESM1_ESM.pdf]

Continuous shear stress alters metabolism, mass-transport, and growth in electroactive biofilms independent of surface substrate transport

Supplementary Information

A-Andrew D Jones, Cullen R Buie*

Scaling Analysis for matching a rotating disk to a packed bed reactor.

To do this, we compare the rotating disc electrode system to membrane bioreactors and packed bed reactors using theories of flow through porous media. The superficial velocity, U_0 is defined as

$$U_0 = \frac{Q}{A}, \tag{1}$$

where Q is the total flow rate and A is the area of the empty reactor. The Reynolds number for the flow is defined as

$$Re_p = \frac{\phi U_0}{(1-\phi)S_v \nu}, \tag{2}$$

where the specific surface area to volume ratio, S_v and porosity ϕ must be measured.[1] The shear stress, τ, on particles in the porous media is then calculated as[1]

$$\tau = \frac{1}{S_v}\frac{\phi}{(1-\phi)}\frac{\mu}{k_p}U_0. \tag{3}$$

The permeability, k_p, while typically measured, can be calculated using the Kozney-Carman equation as,[2]

$$k = \frac{\phi^3}{K(1-\phi)^2 S_v^2}, \tag{4}$$

where $K \approx 5$ is the value commonly used for the Kozney constant.[2] The dimensionless stress similarly found with the fluid inertia is then[1]

$$\frac{\tau}{\frac{1}{2}\rho U_0^2} = \frac{2K}{\phi^2} Re_p^{-1}. \tag{5}$$

Similarity to pipe flow scaled by the porosity and Kozney constant is expected since flow through porous media has been related to flow through packed tubes. [1,3]

Using values from literature, SI Table S1, it is possible to determine a dimensionless shear stress that is common between the two systems.

SI. Table S1 Properties of porous media, reticulated vitreous carbon foam, used as an anode in an Upflow Microbial Fuel Cell from He et al.[4]

ϕ	A [cm^2]	S_v [m^{-1}]	k [m^2]	Q [m^{3}s^{-1}]	U_0 [m s^{-1}]
0.97	26	51.05	0.0779	7.56e-7	2.9e-4

To correlate mass transport with the up-flow microbial fuel cell, we use an expression for mass flux for pipe flow modified appropriately for porous media.

$$N = 0.844 c_\infty D_0 Re_p^{\frac{1}{3}} Sc^{\frac{1}{3}} \left(\frac{D^{-2}}{L}\right)^{\frac{1}{3}}, \tag{6}$$

where the diameter is

$$D = \frac{\phi}{(1-\phi)S_v}. \quad (7)$$

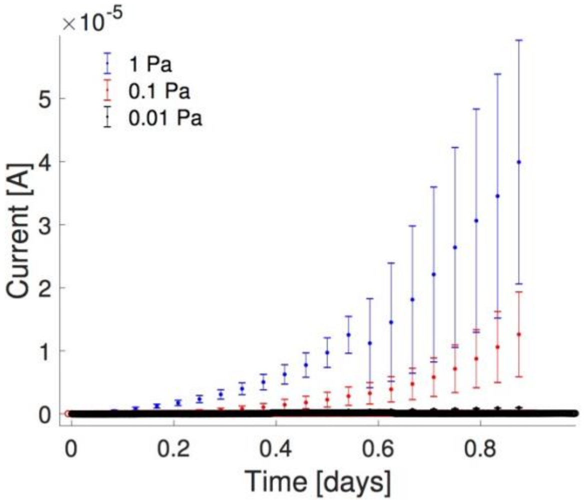

SI Figure S1. The current rises as a function of time following an exponential growth. The doubling times are found as 0.14, 0.12, and 0.26 days for 1, 0.1, (*s.e. n=3*) and 0.01 Pa (*s.e. n = 2*) respectively. This shows that growth rates are increased by increasing shear stress.

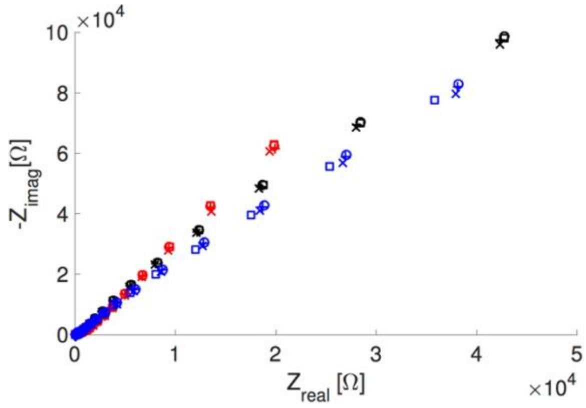

a.

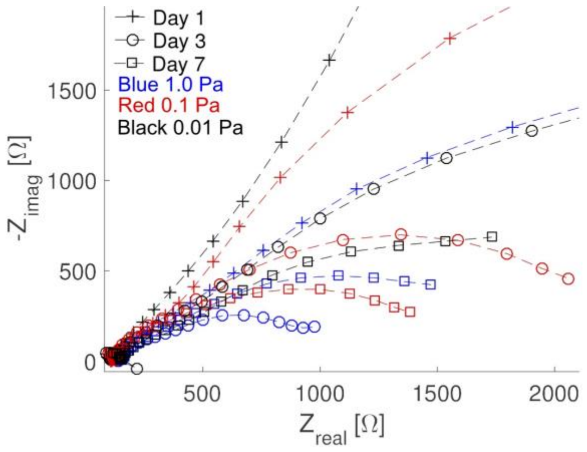

b.

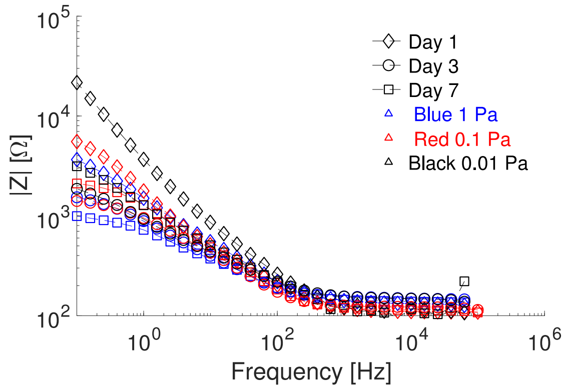

c.

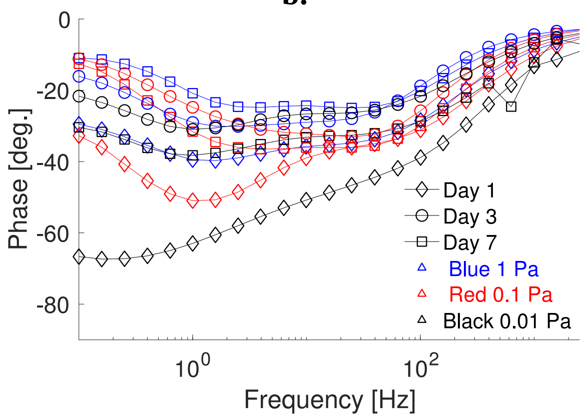

d.

SI Figure S2a. There are subtle differences between each abiotic cell because of differences in conductivity of the background media as described in the Methods. The linear relationship between the imaginary and real parts of the impedance is hypothesized as capacitive since acetate is poorly oxidized on a graphite electrode leading to little current flow. **S2b.** Temporal evolution of electrochemical impedance spectroscopy. The decrease in electron transfer impedance follows the electron diffusion current closely as expected. There is an increase in impedance from day 3 to day 7 of the 1 Pa case but not the other two shear stress cases. The absence of the increase in the 0.1 Pa and 0.01 Pa cases clarifies that the steady state seen in both electron diffusion and chronoamperometry has not started to decrease. The increase in impedance may be due to decrease in redox proteins from loss of or death of cells, decrease in the biofilm pH which may cause a loss of conductivity and or metabolic activity.[5,6] While the curves for day 1 do increase for all three cases, they are not, and should not be, the same as those in **S2a.** because **S2b.** is 24 hours after inoculation. Figure **S2c., S2d.** Bode plot of the data shown in figures **S2a., S2b**. We recognize a similar response to that found by Marsili et al.[7]

Babauta and Beyenal[8] looked at impedance as a function of rotation rate. The rotation rate was changed on impulse and not with regards to continuous shear. A different approach was taken by Bonanni et al., to measure dependence of electron and substrate diffusion as well as substrate advection on limiting current.[9] Their work did not operate near maximum flux and subsequently was not intended to differentiate between the effects of momentum and mass transport.

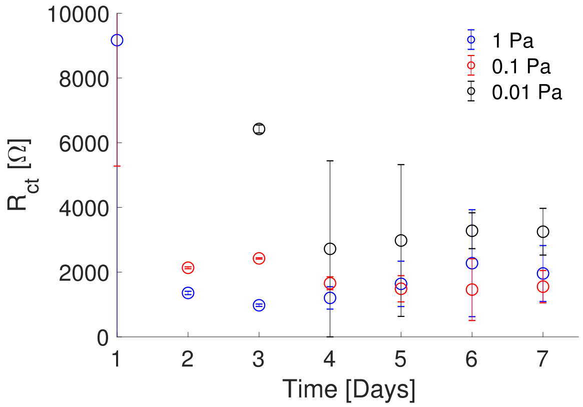

a.

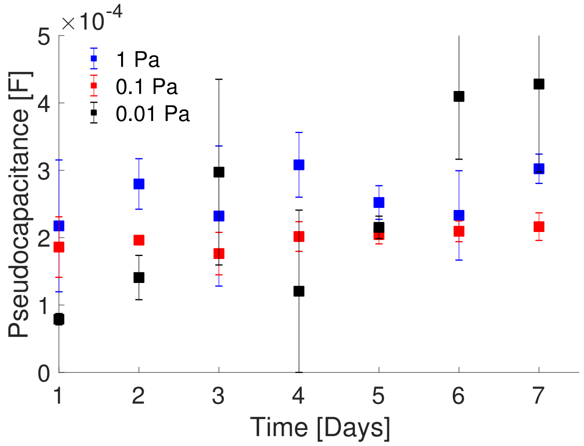

b.

SI Figure S3. Equivalent circuit elements for *G. sulfurreducens* biofilm under shear over time. We fit the impedance data to the simple model in[7] where the solution resistance is in series with a charge transfer resistance in parallel with a constant phase element. The resulting **S3a.** charge transfer resistance shows decay for the 0.01 and 0.01 shear stress cases while an increase in the 1 Pa case and **3b.** the pseudocapacitance shows minor oscillation about a mean (0.26±0.04, 0.20±0.01,0.14±0.24 mF) **Figure 1a.**, or the apparent diffusivity, **SI Figure S6b**.

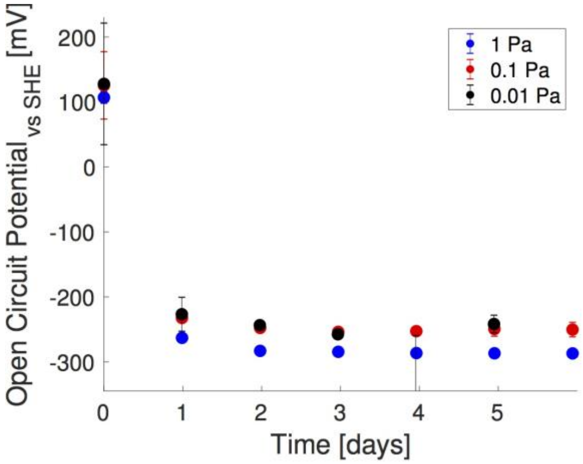

SI Figure S4. The open circuit voltage falls to -266±25 mV vs SHE after one day and is stable throughout the course of the experiment. This value agrees with that found by Bond and Lovley [10] -420 mV vs Ag/AgCl assuming saturated KCl as the filling electrolyte and subsequent studies on *G. sulfurreducens* strain PCA. This is expected, indicating that the bacteria have colonized and are respiring on the electrode.

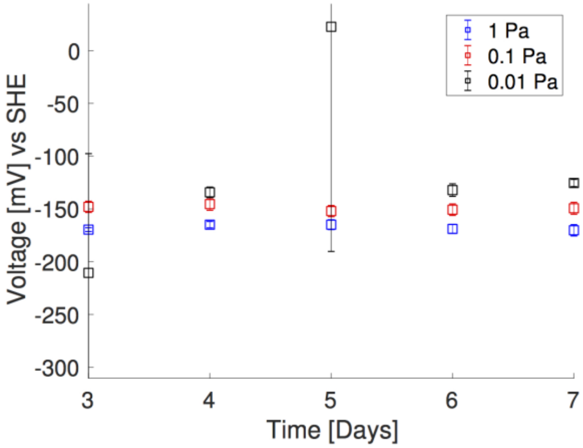

SI Figure S5. The formal potential of the terminal cytochrome is 160±5 mV vs SHE in our work vs 145±10 mV vs SHE by Richter et al., (2009).[11] It is stable within a standard deviation for each condition past day 3. The values were calculated from cyclic voltammograms taken once a day after a 30 min open circuit potential measurement at 2 mV.s^{-1} from the second of 2 cycles.

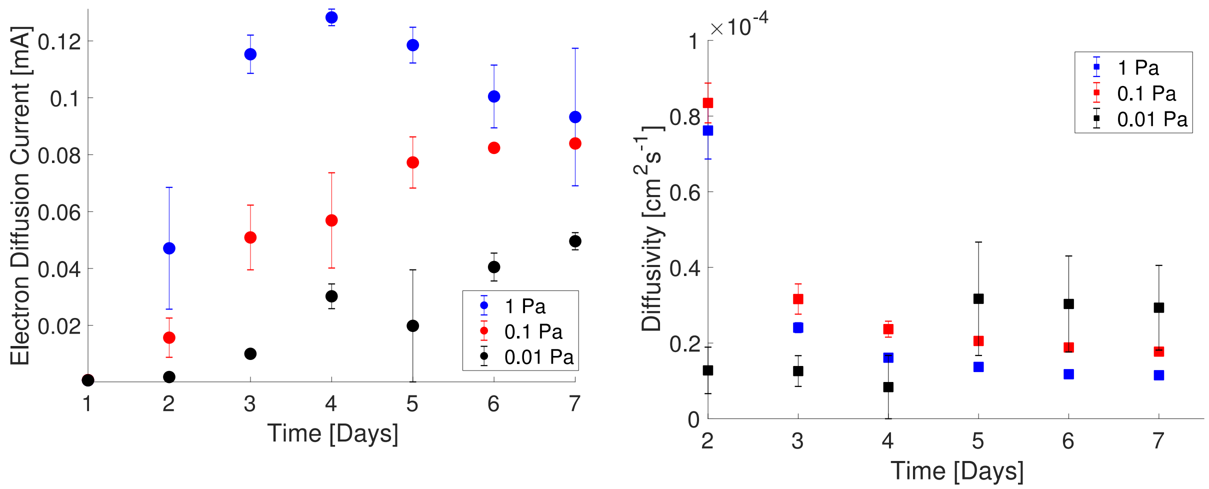

SI Figure S6a. The electron diffusion current was measured as the maximal current generated during a slow (2 $mV.s^{-1}$) cyclic voltammetry scan. Richter et al.,[11] found a maximum electron diffusion current of 0.17±0.005 mA slightly greater than our maximum at the same scan rate. We were not able to quantify the amount of redox mediators present, nor their reaction rate, so we were not able to quantitatively assess the nature of the diffusion current using the expression given by Richter et al.[11] We note that these values are equivalent to the maximum current measured for a given day in Figure 1a.
S6b. In contrast, the apparent diffusivity of the reactive species acetate, displays an exponential decay.

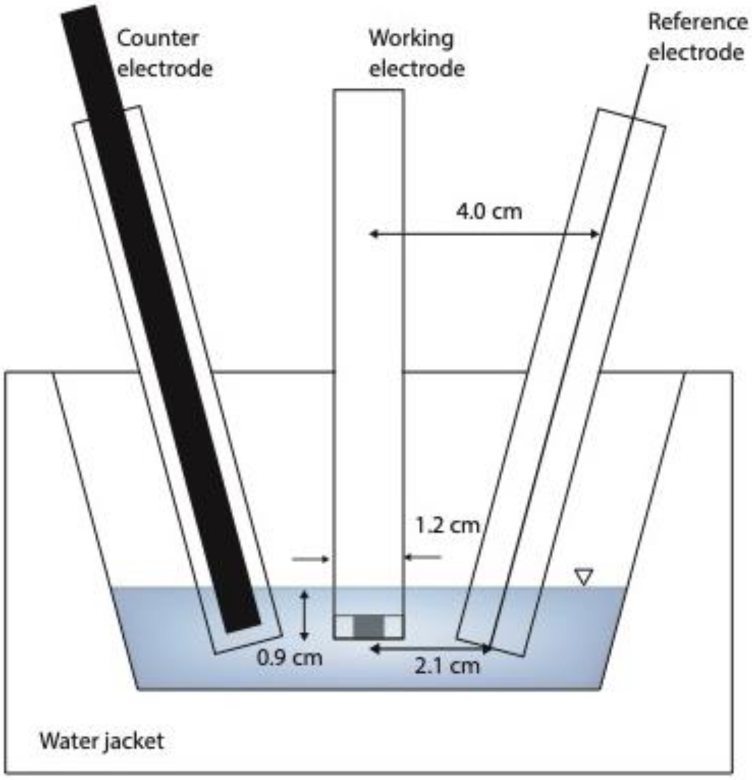

SI Figure S7. Schematic of the electrical elements of the rotating disk system assuming 100 mL of liquid. The height of the liquid is approximately 17.8 mm, not including displacement, The depth the electrode was 9 mm into the solution, the counter and reference electrode were repeatably controlled in

fixed positions by the use of a PTFE gasket that held each glass electrode holder to a fixed position in the neck of the electrochemical cell.

1 Holdich, R. Ch. 3, 21-28 (Midland Information Technology \& Publishing, 2002).

2 Carman, P. Fluid flow through granular beds. *Chemical Engineering Research and Design* **75**, S32-S48, doi:10.1016/S0263-8762(97)80003-2 (1997).

3 Mason, E. A. & Malinauskas, A. *Gas Transport in Porous Media: The Dusty-Gas Model*. (Elsevier Science Publishing Company, INC, 1983).

4 He, Z., Minteer, S. & Angenent, L. T. Electricity generation from artificial wastewater using an upflow microbial fuel cell. *Environmental Science and Technology* **39**, 5262-5267, doi:10.1021/es0502876 (2005).

5 Franks, A. E. *et al.* Novel strategy for three-dimensional real-time imaging of microbial fuel cell communities: monitoring the inhibitory effects of proton accumulation within the anode biofilm. *Energy \& Environmental Science* **2**, 113-119, doi:10.1039/B816445B (2009).

6 Popat, S. C. & Torres, C. I. Critical transport rates that limit the performance of microbial electrochemistry technologies. *Bioresource Technology* **215**, 265-273, doi:10.1016/j.biortech.2016.04.136 (2016).

7 Marsili, E., Rollefson, J. B., Baron, D. B., Hozalski, R. M. & Bond, D. R. Microbial Biofilm Voltammetry: Direct Electrochemical Characterization of Catalytic Electrode-Attached Biofilms. *Appl Environ Microb* **74**, doi:10.1128/AEM.00177-08 (2008).

8 Babauta, J. T. & Beyenal, H. Mass transfer studies of Geobacter sulfurreducensbiofilms on rotating disk electrodes. *Biotechnology and Bioengineering* **111**, 285-294, doi:10.1002/bit.25105 (2013).

9 Bonanni, S. P., Bradley, D. F., Schrott, G. D. & Busalmen, J. Limitations for Current Production in Geobacter sulfurreducens Biofilms. *ChemSusChem* **6**, 711-720, doi:10.1002/cssc.201200671 (2013).

10 Bond, D. & Lovley, D. R. Electricity Production by Geobacter sulfurreducens Attached to Electrodes. *Applied and Environmental Microbiology* **69**, 1548-1555, doi:10.1128/AEM.69.3.1548-1555.2003 (2003).

11 Richter, H. *et al.* Cyclic voltammetry of biofilms of wild type and mutant Geobacter sulfurreducens on fuel cell anodes indicates possible roles of OmcB, OmcZ, type IV pili, and protons in extracellular electron transfer. *Energy Environ Sci* **2**, 506-516, doi:10.1039/B816647A (2009).
